# A novel *GLI3* mutation affecting the zinc finger domain leads to preaxial-postaxial polydactyly-syndactyly complex

**DOI:** 10.1186/s12881-014-0110-9

**Published:** 2014-09-30

**Authors:** Michael Volodarsky, Yshaia Langer, Ohad S Birk

**Affiliations:** The Morris Kahn Laboratory of Human Genetics, National Institute for Biotechnology in the Negev and Faculty of Health Sciences, Ben Gurion University, Beer-Sheva, 84105 Israel; Department of Pediatrics, Shaare-Zedek Medical Center, Jerusalem, Israel; Genetics Institute, Soroka Medical Center, Beer-Sheva, 84101 Israel

**Keywords:** Polydactyly, Syndactyly, GCPS, *GLI3*, Mutation, C2H2, Zinc finger

## Abstract

**Background:**

Polydactyly is a highly common congenital limb defect. Extra digits may appear as an isolated anomaly or as a part of a syndrome. Mutations in *GLI3* have been shown to cause Greig cephalopolysyndactyly, Pallister-Hall syndrome and non-syndromic polydactyly. Genotype-phenotype correlation studies of *GLI3* mutations suggest a model by which mutations in the zinc-finger domain (ZFD) of GLI3 likely lead to syndromic polydactyly. Here we describe a rare case of autosomal dominant heterozygous missense mutation in the ZFD of GLI3 leading to a variable polydactyly-syndactyly complex.

**Case presentation:**

A large Jewish Moroccan family presented with apparently autosomal dominant heredity of bilateral thumb polydactyly in hands and feet combined with post-axial polydactyly type B or type A. Syndactyly was evident in most patients’ hands and feet. Apart from head circumference beyond 90^th^ percentile in some of the affected individuals, none had craniofacial dysmorphism. A novel *GLI3* c.1802A > G (p.His601Arg) mutation was found in all affected individuals.

**Conclusion:**

We demonstrate that a mutation in the ZFD domain of *GLI3* leads to phenotypic variability, including an isolated limb phenotype. Thus, the variability in phenotypes caused by mutations in this master developmental regulator is more profound than has been previously suggested.

**Electronic supplementary material:**

The online version of this article (doi:10.1186/s12881-014-0110-9) contains supplementary material, which is available to authorized users.

## Background

Polydactyly, one of the most common congenital hand/foot malformations encountered in clinical genetics [1,2], can occur as an isolated entity or as part of pleiotropic developmental anomaly syndromes [3]. Post-axial polydactyly is far more common than pre-axial and central polydactyly; occasionally, concomitant syndactyly is seen with some forms of polydactyly [2]. The GLI3 protein is a zinc finger transcription factor expressed early in development [4]. It is required for the specification of dorsal cell types and for suppression of ventral cell types in the forebrain [5]. *GLI3* expression in the anterior half of the limb buds appears to play an important role in suppressing digit formation [5]. These observations, together with the diverse phenotypes resulting from molecular defects in *GLI3*, serve as a classical example of pleiotropy: *GLI3* mutations are known to cause different clinical entities: Greig Cephalopolysyndactyly Syndrome (GCPS) (MIM ID #175700), Pallister-Hall Syndrome (MIM ID #146510), Acrocallosal Syndrome (MIM ID #200990), Pre-axial Polydactyly type IV (MIM ID #174700) and Post-axial Polydactyly type A (MIM ID #174200). The great majority of known *GLI3* mutations cause loss of function [4]*.* Some reports describe *GLI3* mutations as a cause of isolated polydactyly [6], while others consider all *GLI3* mutations as causing polydacyly within the GCPS spectrum [7]. It has been suggested that mutations in different domains of the gene underlie the different evolving phenotypes, and that mutations 5′ to or within the zinc finger domain (ZFD) of GLI3 specifically cause GCPS [6,8]. The classic clinical presentation of GCPS is a triad of polysyndactyly, macrocephaly and hypertelorism [9]. Here we report a new heterozygous missense mutation in the zinc finger domain of *GLI3* leading to a variable phenotype presenting in some cases as an isolated polydactyly-syndactyly complex.

## Case presentation

**Figure 1:**
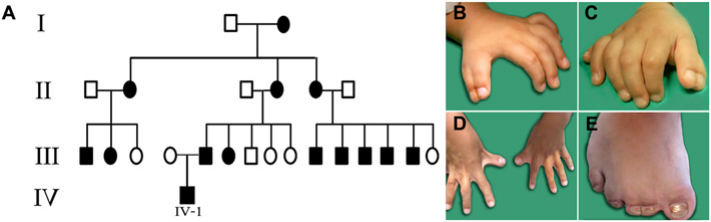
**The clinical phenotype. A**. Family tree of the kindred studied. **B**,**C**. Pre-axial polydactyly combined with post-axial polydactyly (individual IV:1). **D**,**E**. Various hands/feet syndactyly phenotypes of affected individuals (each photograph is of a different affected individual).

A large Jewish Moroccan kindred presented with apparently autosomal dominant heredity of polydactyly (Figure 1A). Affected and unaffected family members underwent thorough clinical and molecular evaluation following Soroka Medical Center IRB approval and informed consent. Phenotypic variability among the 14 affected individuals was evident: most had hands and feet bilateral thumb polydactyly and post-axial polydactyly type B. Syndactyly was found in the feet of all affected individuals and in the hands of most (Figure 1D,E). A single case of thumb polydactyly combined with post-axial polydactyly type A (well developed separated digit) was evident (Figure 1A, IV:1; Figure 1B,C). None of the affected individuals had apparent craniofacial dysmorphism. Occipitofrontal head circumference (OFC) and interpupillary distance (IPD) were measured in 13 (8 affected and 5 unaffected) family members. The measurements were most variable (see Additional file 1). Five patients had an OFC at or above the 90th percentile, while all the healthy individuals showed OFC beneath 90th percentile. As to IPD measurements, 5 of 8 affected as well as 4 of 5 unaffected individuals were above 97th percentile.

### Methods and results

**Figure 2:**
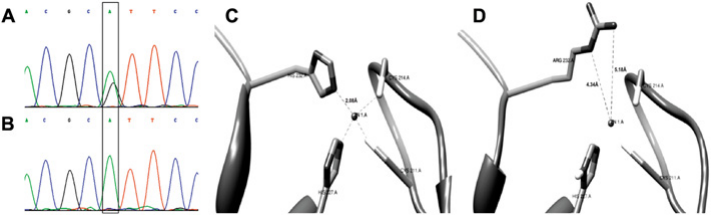
**The** ***GLI3*** **mutation and structural modeling of the predicted mutant protein:** **A**,**B**. Chromatograms illustrating the *GLI3* c.1802A > G heterozygous mutation (A = Affected individual; B = Unaffected individual). **C**,**D**. Structural modeling of the predicted wild type **(C)** and mutant p.His601Arg **(D)** proteins. Four C2H2 comprising residues are shown. Fragmented lines indicate the coordinative bonds between C2H2 residues and the zinc (marked as small spherical shape in the center). Note that in the wild type protein **(C)** the bond distance between the histidine in the upper left side and the metal ion is 2.08 Å, while the shortest bond possible between the zinc and the arginine residue in the predicted p.His601Arg mutant protein is 4.34 Å **(D)**.

Blood samples were obtained from 13 affected and 6 unaffected family members and genomic DNA was extracted by routine techniques. Linkage to genes known to be associated with non-syndromic polydactyly was tested using 2 polymorphic markers flanking each candidate gene. Association of the phenotype with *HOXD13*, ZPA regulatory sequence (ZRS) and *FBLN1* was ruled out (data not shown). Polymorphic markers D7S1526 and D7S691 flanking *GLI3* identified a heterozygous haplotype shared by all affected family members (data not shown). Sanger sequencing of all *GLI3* exons and their flanking exon-intron boundaries and comparison (NCBI BLAST) to the published *GLI3* sequence (GenBank reference number NM_000168.5), identified a single heterozygous missense mutation in exon 12: c.1802A > G, p.His601Arg (Figure 2A,B). This novel mutation was found to segregate within the kindred as expected, demonstrating full penetrance of the phenotype.

The GLI3 ZFD consists of 5 C2H2 type zinc fingers. The histidine to arginine substitution caused by the mutation is of the second histidine within one of the C2H2 zinc fingers comprising this domain. In zinc finger domains, a zinc ion forms four coordinate bonds generating a stable fold [10]. Since the GLI3 601His is one of the four residues directly interacting with the Zn^2+^ ion in a C2H2 zinc finger motif, the p.His601Arg substitution is likely to have functional consequences. Using crystallographic structure of the consensus motif of C2H2 type zinc finger (PDB:3IUF), we used UCSF Chimera software [11] to construct a simplified model illustrating the change in interactions with the Zinc cation. As shown in Figure 2, the mutation is predicted to cause destabilization of the Zn^2+^ ion, as the stable coordinative bond of histidine 232 (comparable to GLI3 histidine 601) with the metal ion (Figure 2C) is replaced in the mutated protein by a much longer unrealized bond with an arginine residue (Figure 2D). We speculate that the p.His601Arg mutation might alter the ability of GLI3 to bind its downstream targets within the SHH pathway, such as the posterior *Hoxd* genes [12].

## Conclusions

In the kindred we describe, the OFC and IPD findings possibly stem from intrafamilial variability. The craniofacial manifestations of GCPS are highly variable and not all patients with GCPS have obvious macrocephaly [9] as observed in the family described here. In fact, the mild end of the GCPS spectrum is a continuum with isolated polydactyly [7]. Thus, in some cases there is unclear clinical delineation of GCPS versus non-syndromic polydactyly. The effects of truncating mutations (leading to loss of functional regions) on digit number and identity were largely discussed in the context of GLI3′s role as mediator of the hedgehog pathway, whereas connection between defect in the DNA binding domain (DBD) and the phenotype of our pedigree is more elusive. Besides the posterior *Hoxd* genes, other factors known to be downstream of *GLI3* are *FGF8*, *FGF4*, *GREM1*, *HAND2* and *JAG1* [12]. It is plausible that changes in expression patterns of these GLI3 targets (as a result of an obstructed DBD) can contribute to the polydactyly phenotype. Both the precise mechanism through which the specific mutation leads to the disease phenotype, and the molecular mechanisms underlying the familial phenotypic variability are yet to be elucidated.

In summary, we describe a large kindred with a novel heterozygous *GLI3* ZFD domain missense mutation leading to polydactyly-syndactyly complex. The phenotype described is within the wide range of phenotypic spectrum of GCPS established by Biesecker [13]. With recent emerging evidence of *GLI3* mutations causing non-syndromic limb defects [14,15], our data highlight the fact that a *GLI3* mutation within the ZFD domain can cause a mild form of GCPS with no prominent facial dysmorphism. This report strengthens the need to screen for *GLI3* mutations in patients with polydactyly-syndactyly phenotype, even when syndromic features are not evident.

### Consent

Written informed consent was obtained from the patients for publication of this Case report and any accompanying images.

## Additional file

Additional file 1:
**Clinical data.** Gender, affected (+/−), age, OFC, IPD and malformations data of family members.
