# Supplementary material for: A novel GLI3 mutation affecting the zinc finger domain leads to preaxial-postaxial polydactyly-syndactyly complex
Source: BMC Med Genet. 2014 Sep 30;15:110. doi: 10.1186/s12881-014-0110-9 (PMC4256830; doi:10.1186/s12881-014-0110-9)
Supplement: Additional file 1: — Clinical data. Gender, affected (+/−), age, OFC, IPD and malformations data of family members. [file 12881_2014_110_MOESM1_ESM.pdf]

Clinical Data

Pt.	Gender	Affected	Age (year)	OFC (cm)	% for age*	IPD (mm)	% for age*	Syndactyly**	Polydactyly**
I:1	F	+						Simple, Incomplete Hand+foot	Postaxial, type B Preaxial type 4
II:1	F	+						Simple, Incomplete Hand+foot	Preaxial type 4
II:2	F	+	53	56.3	75	6.6	97	Simple, Incomplete Hand+foot	Postaxial, type B Preaxial type 4
II:3	F	+						Simple, complete, Foot	Preaxial type 4
III:1	M	+						Simple, Incomplete Hand+foot	Preaxial type 4
III:2	F	+						Simple, Incomplete Hand+foot	Preaxial type 4
III:3	F	–	30	56.4	78	6.5	97	–	–
III:4	M	+	31	59	91	6.8	97	Simple, Incomplete Hand+foot	Postaxial, type B Preaxial type 4
III:5***	F	+	30	56.2	40	6.8	97	Simple, Incomplete Hand+foot	Postaxial, type B Preaxial type 4
III:6	M	–	22	56.4	50	6.7	95	–	–
III:7	F	–	19	51.8	5	5	3	–	–
III:8	F	–	17	54.6	40	6.3	95	–	–
III:9	M	+	6	50.7	50	5.4	75	Simple, complete, Foot	Preaxial type 4
III:10	M	+	11	57.6	95	6.1	80	Simple, complete, Foot	Preaxial type 4
III:11	M	+	2	53.1	95	5.3	85	Simple, Incomplete Hand+foot	Postaxial, type B Preaxial type 4
III:12	M	+						Simple, complete, Foot	Preaxial type 4
III:13	M	+	22	58.9	90	6.6	97	Simple, Incomplete Hand+foot	Postaxial, type B Preaxial type 4
III:14	F	–	25	57.6	75	6.5	97	–	–
IV:1	M	+	4	52.4	90	5.8	97	Simple, Incomplete Hand+foot	Postaxial, type A Preaxial type 4

OFC – occipitofrontal head circumference.

IPD – Interpupillary distance.

M – male, F – female.

*Percentage was calculation by Growth references from 'Growth References Third Trimester to Adulthood' published by Greenwood Genetic Center 1998.

** All cases are bilateral.

*** Mild mental retardation.
